# The effectiveness of the Congo Red Dot paper test in hypertensive disorders of pregnancy: A systematic review and meta-analysis

**DOI:** 10.3389/frph.2023.1120937

**Published:** 2023-02-13

**Authors:** O. P. Khaliq, W. N. Phoswa, J. Moodley

**Affiliations:** ^1^ Department of Obstetrics and Gynaecology and Women’s Health and HIV Research Group, Nelson R Mandela School of Medicine, University of KwaZulu-Natal, Durban, South Africa; ^2^ Department of Life and Consumer Sciences, University of South Africa, Science Campus, Roodepoort, South Africa

**Keywords:** Congo-red-dot paper test, detection, hypertensive disorders of pregnancy (HDP), diagnosis, accuracy

## Abstract

**Background:**

Congo Red Dot Paper Test (CRDPT) appears to be a simple, cost-effective, non-invasive diagnostic tool for hypertensive disorders of pregnancy (HDP). The main objective of the study is to assess the effectiveness of CRDPT in detecting HDP.

**Methods:**

This is a systemic review and meta-analysis of published studies on the effectiveness of CRDPT in the detection of HDP. The study was conducted in line with the PRISMA-DTA guidelines. The PICOS framework was used to search for relevant articles using Medline, PubMed, Google Scholar, Web of Science, and the Cochrane Library databases. The articles were screened against a set of inclusion and exclusion criteria and analysed using the Review Manager 5.4 software.

**Results:**

A title, abstract and full article screening was conducted on 18,153 potential articles based on the inclusion and exclusion criteria. The screening yielded five articles for meta-analysis. The total number of normotensive pregnant women (*n* = 3,380) in the included studies was five times higher than the total number of women with pre-eclampsia (*n* = 535). A difference between the HDP and normotensive group was noted. This is indicated by a significantly decreased in the effectiveness of CRDPT in detecting HDP as compared to normotensive group [Risk Ratio (RR) = 6.32 (2.17, 18.43) *p* < 0.00001]. The included studies had a high nature of heterogeneity (*I*^2^ = 98%, *p* < 0.00001) partially due to different study designs included in the analysis and different regions where studies were conducted given that none of these studies were conducted in African countries where HDP is prominent.

**Conclusions:**

According to results generated from 5 studies in this meta-analysis, it was found that CRDPT might not be effective in the detection of hypertensive disorder of pregnancy. Moreover, more research, especially in African women where hypertensive disorders of pregnancy are prevalent, are re-quired to ascertain these findings.

**Systematic Review Registration:**

https://www.crd.york.ac.uk/prospero/display_record.php?ID=CRD42021283679, identifier: CRD42021283679.

## 1. Introduction

Pregnant women worldwide are at a risk of developing complications, in particular, hypertensive disorders of pregnancy; the most common being pre-eclampsia (PE). Pre-eclampsia affects 5%–8% of pregnancies worldwide, leading to substantial complications such as foetal and maternal morbidity and mortality (1). In low-and-middle-income countries (LMICs), especially those in sub-Saharan Africa, hypertensive disorders of pregnancy (HDP) account for 16% of maternal deaths (2–4). An alarming 18% of maternal deaths in South Africa (SA) result from HDP (5) The high incidence in SA may be attributed to the high burden of HIV, especially in pregnant women (6). Studies have reported an association between HIV treatment and the pathogenesis of pre-eclampsia in SA (6–8).

The International Society for the Study of Hypertension in Pregnancy (ISSHP) classified HDP into different types: white coat hypertension, masked hypertension, Haemolysis, elevated liver enzymes, low-platelet count (HELLP) syndrome, chronic hypertension (CH), gestational hypertension (GH), pre-eclampsia, pre-eclampsia with severe features and eclampsia (9). Pre-eclampsia and eclampsia are the principal causes of maternal and foetal mortality among the other conditions (5).

The incidence of maternal and foetal deaths due to HDP are evidently high and it is believed that early diagnosis of these conditions may decrease maternal and perinatal mortality. However, the diagnosis and distinction among all HDP classes have been challenging, especially in LMICs. The maternal and foetal outcomes of the different classes of HPD and the level at which the classes affect pregnant women differ. Eclampsia and PE are more aggressive compared to the other categories of HDP.

Pre-eclampsia is commonly defined and diagnosed as elevated blood pressure (>140/90 mmHg) commencing at >20 weeks of pregnancy with subsequent protein (300 mg per 24 h or 1 + dipstick) in the urine (10). According to the latest guidelines from ISSHP, PE may occur in the absence of proteinuria with high blood pressure in combination with disorders such as: renal disease, liver disorders, pulmonary oedema, thrombocytopenia, or fetal growth restriction (11). Pre-eclampsia affects 5%–8% of all pregnancies worldwide (1). Approximately 50,000–60,000 maternal deaths occur yearly due to PE (1). Of all the different classifications of HDP, PE has the highest prevalence in SA in which it complicates 14.8% of all pregnancies (12). The risk features of PE include first pregnancy, obesity, advanced maternal age (>35), a family history of HDP, African ancestry, and paternal influence (if the father of the baby has a history of PE or born from a mother who had PE or fathered a pre-eclamptic pregnancy previously). Currently, PE has no cure, the only known way is iatrogenic termination of pregnancy since the aetiology is thought to arise during placentation (13). Symptoms of PE include, Severe headaches, oedema, especial in the facial area, blurry vision, abdominal pain, difficulty breathing, tension or confusion, and seizures (14).

Since an angiogenic imbalance also characterises PE, angiogenic markers have been suggested as early predictors of PE (15). However, this method of diagnosis is difficult to implement in LMICs due to limited resources (financial, technical) and possibly contradictory results in local studies (11, 16, 17).

Other categories of the HDP that commonly occur are GH (>20 weeks gestation) and CH (<20 weeks gestation) in the absence of proteinuria (18). Similar to CH, GH may progress to PE but the mechanism is yet to be elucidated (19). Gestational hypertension affects 10%–15% of all pregnancies worldwide (20). Also, similar to CH, GH results in adverse maternal and foetal outcomes. The similarities noted among the different HDP classes make it difficult for health workers and clinicians to make a definitive diagnosis. Pre-eclampsia for instance, may easily be misdiagnosed and only discovered when the disorder has rapidly progressed to eclampsia (seizures). It is therefore imperative that these conditions are detected in early pregnancy to eliminate adverse outcomes.

Recently, a way to solve this predicament was found by the introduction of a new way to test for PE and most importantly, to distinguish it from other classes of HPD. The CRDPT was initially introduced by Buhimschi et al. (21). This test was used to detect misfolded proteins in the urine of women suspected to have HDP. Misfolded proteins have a high binding affinity for CRD. Thus the CRDPT was manufactured and its effectiveness tested in women with HDP (21). In the study by Buhimschi et al*.*, prominent levels of congophillia were discovered in the urine of women with PE, indicating a high level of misfolded proteins compared to controls and triggered an interest in the use of the CRDPT to detect PE or superimposed PE in CH.

Several ways to diagnose PE have been proposed previously (21). However, most tests were time-consuming, expensive, and some reports showed contradictory results (22). Besides elevated BP, the commonly practiced way of diagnosing PE is through detecting proteinuria. Buhimschi et al. reported that proteinuria indicated that PE was a protein configuration disorder (21). Rood et al. reported using the CRDPT to distinguish PE from the other HDP classes (22). The advantages of CRDPT are that they are quick and easy to perform, accurate, non-invasive and cost-effective (22). This theory was tested in a group of pregnant women with HDP. Congophilia was found in 12% of pregnant women with a tentative diagnosis, 58% of patients admitted for PE confirmed positive on the CRDPT and only 10% did not have congophilia resulting in a change in diagnosis (10). These results demonstrate that the CRDPT is effective and has the potential to be used as an accurate and early predictor of PE. Furthermore, it was reported that this test can be used to rule out PE in women with other categories of HPD (10).

In India, a group of researchers conducted a study using the Rood et al. protocol (22). The study population consisted of women with early-and late-onset PE. These sub-divisions of PE are based on gestational age. Early-onset PE occurs at ≤34 weeks of gestation while late-onset occurs at ≥34 weeks. Studies have reported that early-onset PE results in severe features of PE compared to late-onset PE (23, 24). Nonetheless, the CRDPT indicated that misfolded proteins were higher in late-onset PE compared to early-onset PE and the normotensive group (25). These findings designate the variance in pathology of the two classes of PE (26). In contradiction to results reported thus far, Dobert et al. reported that the CRDPT results were “poor” in women between 35 and 37 weeks of gestation (27).

Most studies report positive results on the effectiveness of CRDPT. However, very little has been done in this area of research since no studies have been published in Africa where HDP increases maternal and foetal morbidity and mortality. Table 1 summarizes all prospective studies in different countries that used the CRDPT to diagnose HDP. This table includes the different authors, the year of publication, country, the study population, ethnicity, study design, results, and conclusions. The purpose of this table is to indicate the number of studies that used the test globally and the results reported. In addition, the systematic review aims to conduct a meta-analysis on the studies listed in Table 1 to investigate whether the test is effective. This summary will also reveal areas that require further research with respect to the CRDPT.

**Table 1:** Search strategy.

Database	Search Items	Number of articles	Date
PubMed	https://pubmed.ncbi.nlm.nih.gov/?term=Congo+red+dot+test%2C+pre-eclampsia	3	28 June 2021
EBSCOhost	https://web.b.ebscohost.com/ehost/results?vid=41&sid=6216e251-64b1-46be-8127-cc548e72b8e2%40sessionmgr101&bquery=Congo+Red+Dot+Paper+Test+in+Hypertensive+Disorders+of+Pregnancy&bdata	17,491	27 June 2021
Google scholar	https://scholar.google.com/scholar?hl=en&as_sdt=0%2C5&q=Congo+red+dot+test+in+Pre-eclampsia&btnG=	608	28 June 2021
Science direct	https://www.sciencedirect.com/search?qs=Congo%20Red%20Dot%20Paper%20Test%20in%20Hypertensive%20Disorders%20of%20Pregnancy&offset=50	51	27 June 2021

## 2. Objective

To evaluate the accuracy of the Congo Red Dot Paper Test in diagnosing hypertensive disorders of pregnancy.

## 3. Methods

recommendations of the Preferred Reporting Items for Systematic Reviews and Meta-analysis for Protocols (PRISMA-DTA) guidelines 2020 (27). The results were reported based on the PRISMA 2015 (28), statement and article screening and the selection process was demonstrated through a PRISMA-*P* flow diagram. Furthermore, the current protocol has been registered with the International Prospective Register of Systematic Reviews (PROSPERO): CRD42021283679.

## 4. Eligibility

### 4.1. Study design

A systematic review and meta-analysis of a diagnostic test accuracy that is inclusive of randomized control trials, cohorts, and matched cohorts with a defined population and diagnostic test used. While, observational studies, reviews, case studies, and animal studies were excluded.

### 4.2. Participants

Normotensive women (*n* = 3,380).

Women with Hypertensive disorders of pregnancy (*n* = 535).

### 4.3. Intervention (diagnostic accuracy test (DTA))

Congo Red Dot Paper Test.

### 4.4. Comparator

Normotensives.

### 4.5. Outcomes

Hypertensive disorders of pregnancy, gestational hypertension, normotensive, pre-eclampsia, pregnancy induced hypertension.

### 4.6. Inclusions criteria

a. Journal articles presented in the English language.
b. Full-text Matched cohort, Cohort, cross-sectional studies, randomized control trials primary literature in English text, published between 2014 and 2022 were included
c. Evidence from published global randomized control trial, matched cohort, and cohort studies with hypertensive disorders of pregnancy-related outcomes/complications, and all of the criteria defining the effectiveness of the Congo Red Dot Paper Test in hypertensive disorders of pregnancy

### 4.7. Exclusion criteria

a. Non-English studies,
b. Studies without the outcomes of interest as objectives.
c. Case reports, expert opinions and review/meta-analysis.
d. Evidence published before the year 2014.

### 4.8. Search strategy

The following databases were searched for eligible studies: PubMed, EBSCOhost, Google scholar, and Science direct. Manual searches through EBSCOhost, Google Scholar and science direct were used and free text searches were used to search the eligible articles which were saved to the citation manager EndNote X7 (Thomson Reuters). Medical subject headings (MeSH) such as “((((((“pregnant women”[MeSH Terms] OR (“pregnant”[All Fields] AND “women”[All Fields]) OR “pregnant women”[All Fields]) AND ((“congo red”[MeSH Terms] OR (“congo”[All Fields] AND “red”[All Fields]) OR “congo red”[All Fields]) AND dot[All Fields] AND (“paper”[MeSH Terms] OR “paper”[All Fields]) AND (“research design”[MeSH Terms] OR (“research”[All Fields] AND “design”[All Fields]) OR “research design”[All Fields] OR “test”[All Fields]))) OR crdpj[All Fields]) AND normotensive[All Fields]) AND (hypertensive[All Fields] AND (“disease”[MeSH Terms] OR “dis-ease”[All Fields] OR “disorders”[All Fields]) AND (“pregnancy”[MeSH Terms] OR “pregnancy”[All Fields]))) OR (“hypertension, pregnancy-induced”[MeSH Terms] OR (“hyper-tension”[All Fields] AND “pregnancy-induced”[All Fields]) OR “pregnancy-induced hypertension”[All Fields] OR (“gestational”[All Fields] AND “hypertension”[All Fields]) OR “gestational hypertension”[All Fields])) OR (“hypertension, pregnancy-induced”[MeSH Terms] OR (“hypertension”[All Fields] AND “pregnancy-induced”[All Fields]) OR “pregnancy-induced hypertension”[All Fields] OR (“hypertension”[All Fields] AND “pregnancy”[All Fields]) OR “hypertension in pregnancy”[All Fields])” were used. This software was used to remove duplicates. The title and abstracts of the articles remaining after exclusion of duplicates were assessed for eligibility according to the inclusion and exclusion criteria.

### 5.9. Study selection

The full text of all potentially eligible studies was reviewed by two independent re-viewers (OPK and WNP), and any disagreement between reviewers with respect to eligible studies for inclusion in the analysis were assessed for more eligible studies. Initially, studies were screened by the titles, abstracts, keywords, and synonyms then followed by the identification of the full-text articles. Where discrepancies arose between two authors (OPK, WNP), a third author (JM) screened such studies, and a consensus was reached through discussion. EndNote X7 (Thomson Reuters) was used to manage extracted data items, including saving relevant and excluded studies with reasons. Importantly, reference lists of included studies were screened to confirm that no relevant studies were left out. Studies meeting the inclusion criteria were then subjected to data collection, critical appraisal, risk, and quality evaluation.

The studies that were included in the review had the following characteristics: Pregnant women of any age, with no other pregnancy complications and diagnoses of hyper-tensive disorders of pregnancy (HDP). The studies also had to have a pregnant– non-HDP or control group. Observational studies, cohort, cross sectional studies and randomized control trials were included. The effectiveness of Congo Red Dot Paper Test (CRDPT) had to be tested. Studies that were excluded lacked the testing of the effectiveness of CRDPT in controls (normotensives) and experimental group (HDP), reviews, comments, dissertations, books, abstracts, conferences, and articles that were not yet published. Studies that included non-pregnant women as controls were excluded. Articles where the odds ratio (OR) and confidence intervals (95%) cannot be determined, were excluded.

## 5. Data abstraction, data analysis, and quality assessment

A table was used to collect background information and process the data items from each selected article. To confirm that all the relevant information regarding significant aspects of the study were gathered, a data charting form was created, piloted, and updated.

## 6. Risk of bias and quality assessment

A study classified as high risk of bias indicates low confidence that the results reflect the true treatment effect. The quality and risk of bias of selected articles was performed by both reviewers (WNP and OPK) using Cochrane risk of bias tool (28). This tool takes into consideration the following, random sequence generation, allocation concealment blinding of participants and personnel, blinding of results assessment, incomplete outcome data, and selective reporting.

## 7. Evaluation of quality of evidence

The quality and scientific evidence of the selected articles was determined using the Grading of recommendations assessment, development and evaluation (GRADE) tool to assess risk of bias (29), inaccuracy, variability and indirectness, and placed into one of four categories, ranging from very low (where the true effect is probably markedly different from the estimated effect) to very high (that which was observed is most likely to be true). Randomised controlled trials without limitations therefore, provided the most relevant evidence, with observational studies falling into the lowest category, given the potential for confounding. The strength of the recommendations identified from these articles were evaluated according to the quality of evidence presented. The following authors (WNP and OPK) independently evaluated the quality of the articles using the data extraction tool, with consensus on disagreement being achieved with the assistance of another author (JM).

## 8. Data analysis

• The data were analysed using Review Manager (RevMan) 5.3 software.
• The generic inverse variance method was used for meta-analysis of both, individually and cluster randomised trials to estimate the effect size from risk ratio (RR) and relative confidence intervals (CI)s. In a case where we did not find at least two studies to produce a single estimate of the effect of intervention. We calculated the RR, and 95% CIs by computing the number of events and the number of patients from both control and HDP groups.
• Statistical heterogeneity between studies were evaluated by *I*^2^ statistic and classified as low if *I^2^* < 20% or moderate if *I^2^* > 50%, the fixed effect model to estimate RR and relative confidence intervals. Statistical significance for effect estimates was set at *p* < 0.05.

## 9. Results

### 9.1. Literature search

**Figure 1:**
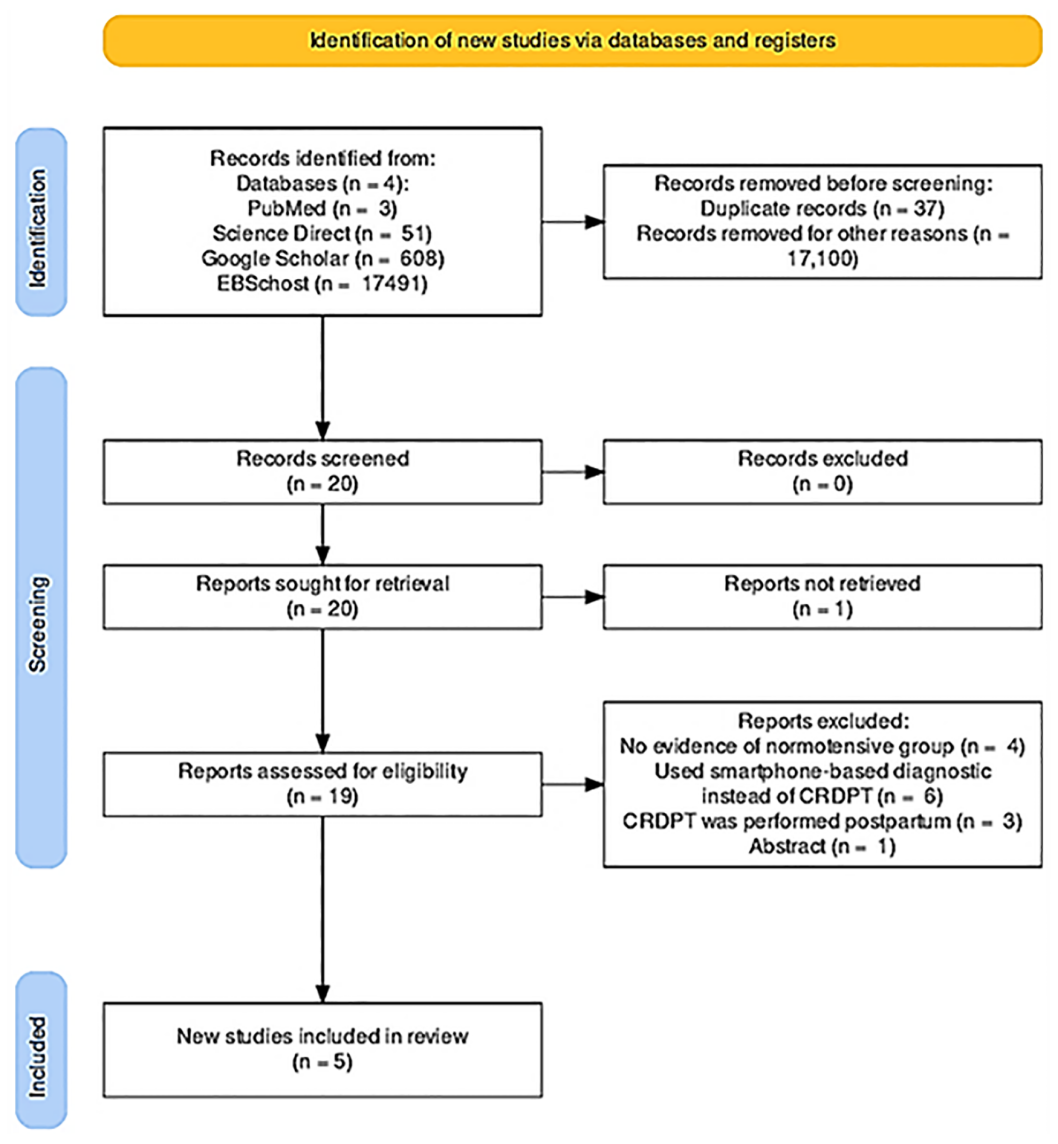
PRISMA flow diagram of the study selection process.

We identified 18 153 studies through PubMed central, Google Scholar, EBSCOhost, and ScienceDirect (Table 1 of Supplementary file S1). The literature search yielded 18,153 articles through our databases (Figure 1). After duplicates were removed (*n* = 160), we screened 1,000 studies for eligibility and excluded 980 studies. We then performed full text article screening in 20 articles and excluded 15 articles with the following reasons: some lacked evidence of control group (normotensive) (=5), others used smartphone-based diagnostic instead of CRDPT (*n* = 6), and in others CRDPT was performed postpartum (*n* = 4). Other studies were review articles, and others only focused on the retention rate of misfolded proteins in HDP vs. normotensives and did not focus on the effectiveness of the CRDPT.

### 9.2. Characteristics of eligible studies

The characteristics of these included studies are summarized in (Table 2). These studies were performed from 2009 to 2021 with sample sizes ranging from 46 to 2094. The included studies were conducted in the following countries: One study from the United States (21), Israel (31), India (32), Bangladesh and Mexico (33), United Kingdom (26). The ancestry of the participants enrolled in the studies were (Caucasian, African, Indian, His-panic, and Mixed race) (Table 2).

**Table 2:** Summary of all the studies that used the Congo Red Dot test to Diagnose Hypertensive Disorders of Pregnancy.

Author	Country	Study Title	Study design	Ethnicity	Aim	Sample Size	Gestational Age	Conclusion
[20]	USA	Assessment of global protein misfolding load by urine "Congo Red Dot" test for diagnosis and prediction of outcome in women with preeclampsia (PE)	Cohort	Caucasian	Design and validate a diagnostic and prognostic test for PE based on urine congophilia as a measure of global protein misfolding load in pregnancy.	HDP:234 Normotensive: 98	20-40 weeks	Assessment of global protein misfolding load by CRR is a simple diagnostic test for PE and for prediction of IND, an important contributor to preterm birth
[31]	Israel	Can staining of damaged proteins in urine effectively predict preeclampsia? Fetal diagnosis and therapy.	Cohort	Black	To assess Congo red urine test in the first trimester for preeclampsia (PE) prediction	PE: 105 Normotensive: 537	26-41 weeks	This study confirms the presence of urinary congophilia in Indian pregnant women with preeclampsia. Furthermore, our study shows that urinary congophilia is not affected by clinical variables like gestational age of onset, severity, superimposition by eclampsia and complication by intrauterine growth restriction and intrauterine death. Urinary congophilia can be used to differentially identify preeclamptic pregnant women from normotensive pregnant women.
[21]	USA	Congo Red Dot Paper Test for Antenatal Triage and Rapid identification of Pre-eclampsia	Prospective cohort	White, Hispanic and black	To determine the diagnostic performance of a paper - based point of care test detecting urine congophilia for rapid triage and diagnosis of PE.	HDP: 112 Normotensives: 234	20-39 weeks	The CRD Paper Test is a simple, non-invasive, "sample-in/answer-out" point-of-care clinical tool for rapid identification of PE.
[32]	India	Congo red dot test in the early prediction and diagnosis of pre-eclampsia in a tertiary health care centre in India. Pregnancy Hypertension	Prospective cohort	Indian	To determine the diagnostic performance of a paper - based point of care test detecting urine congophilia for rapid triage and diagnosis of PE.	HDP: 56 Normotensives: 322	10-34 weeks	CRD test was not only effective in predicting pre-eclampsia but was also useful in differentiating between pre-eclampsia and other forms of hypertension, as well as early onset and late onset pre-eclampsia, with positive predictive value of 80.36% and negative predictive value of 92.86%
[33]	Bangladesh and Mexico	Congo red test for identification of preeclampsia results of a prospective diagnostic case-control study	Prospective diagnostic case-control	Bangladesh and Mexican	We evaluated a beta prototype of a point-of-care test for the identification of urine congophilia in women	HDP:204 Normotensive: 205	34- 39 weeks	The point-of-care test for detection of urine congophilia, is a promising tool for rapid identification of preeclampsia
[26]	UK	Screening for late preeclampsia at 35-37 weeks by the urinary Congo-red dot paper test.	Prospective observational study	White, Black, south Asian, east Asian, Mixed Race	To determine the performance of the urinary Congo-red dot paper test at 35-37 weeks' gestation in the prediction of delivery with PE at 2 and < 2 weeks after assessment.	PE: 46 Normotensive: 2094	35- 37 weeks	The performance of the urinary Congo-red dot paper test at 35-37 weeks' gestation in the prediction of PE is very poor

The primary disease in all the studies was hypertension in pregnancy. The patients were divided into hypertensive disorders of pregnancy (HDP) (e.g., gestational hypertension, superimposed pre-eclampsia, pre-eclampsia, chronic hypertension) and normotensive group and the studies were conducted between the two groups (HDP vs. normo-tensives). The study design types were as follows: 1 Cohort study (31), 1 prospective observational study (26), 1 prospective study (20), 2 prospective cohort (21, 32), 1 prospective diagnostic case-control (33).

### 9.3. Effectiveness of Congo red dot paper test (CRDPT)

Five studies reported that CRDPT can be used to verify HDP. In a study by Bracken et al., 204 were pre-eclamptic and 205 normotensive women, the CRDPT was found to be effective in 155 and 50 respectively (30). In a study by Dobert et al. which included 46 pre-eclamptic and 2,094 pregnant normotensives, the CRDPT was found to be effective in 2 and 4 respectively (27). Rood et al. included 112 pre-eclamptics and 234 normotensives, the CRDPT was found to be effective in 96 and 138 respectively (22). Sailakshmi et al. reported that in 68 pre-eclamptic and 310 normotensives, the CRDPT was found to be effective in 46 and 11 respectively (32). Sammar et al. had 105 pre-eclamptic women and 537 normotensives recruited, CRDPT was found to be effective in 48 and 158 women respectively (31) (Table 2).

## 10. Meta-analysis

**Figure 2:**
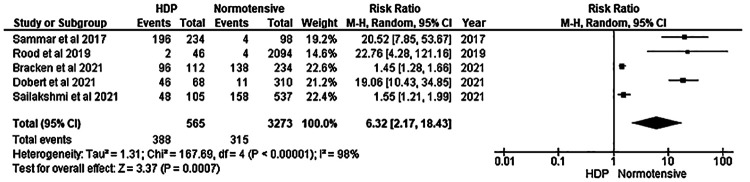
Forest plot indicating the effectiveness of Congo Red Dot Paper Test.

The meta-analysis on the effectiveness of CRDPT is displayed in Figure 2. Only five studies were included in our meta-analysis.

**Figure 3:**
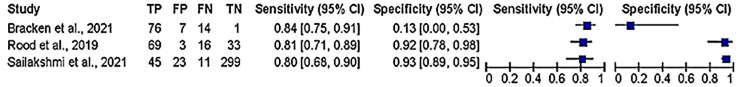
Evaluating sensitivity and specificity of CRDP test in detection of hypertensive disorder of pregnancy.

Our meta-analysis revealed a significant difference between the HDP and normotensive group. This is noted by a significantly decreased in the effectiveness of CRDPT in detecting HDP as compared to normotensive group [(Risk Ratio (RR) = 6.32 (2.17, 18.43) *p* < 0.00001]. However, the included studies had a high nature of heterogeneity [*I*^2^ = 98%, *p* < 0.00001] partially due to different study designs included in the analysis and different regions where studies were conducted given that none of these studies were conducted in African countries where HDP is prominent. More importantly, sensitivity analysis was conducted using one study exclusion approach, and the result showed a minimal change in the level of heterogeneity [[RR = 4.18 (1.67, 10.46) *p* < 0.00001] [*I*^2^ = 96%, *p* < 0.00001] (Figure 3).

### 10.1. Diagnostic accuracy of test

**Figure 4:**
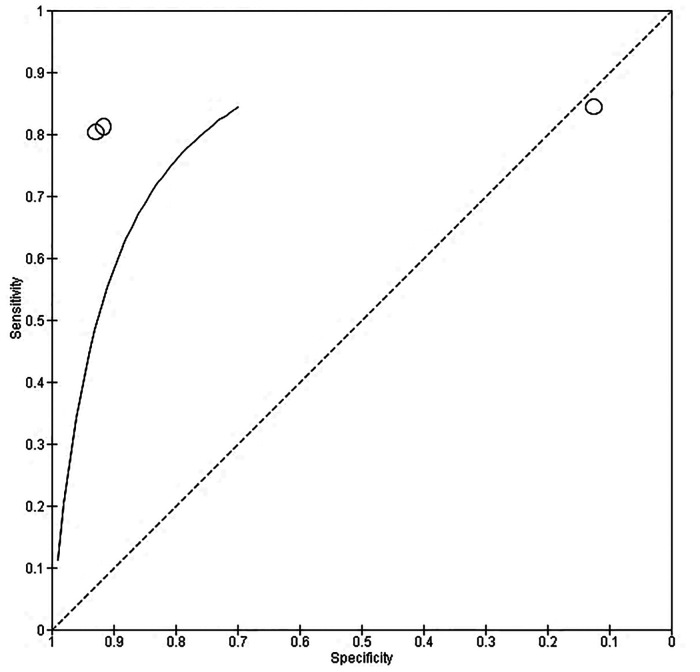
Receiver Operator Characteristic (ROC) curves obtained for CRDP test.

About three studies reported enough data on the parameters of accuracy (sensitivity and specificity) to be pooled out for accuracy of the of the CRDPT test in detecting HDP, and the results are presented in Figure 4. Furthermore, Receiver Operator Characteristic (ROC) curves was used to assess the accuracy of CRDP test in detecting HDP (Figure 4).

### 10.1.1. Quality of studies and the risk of bias assessment

**Figure 5:**
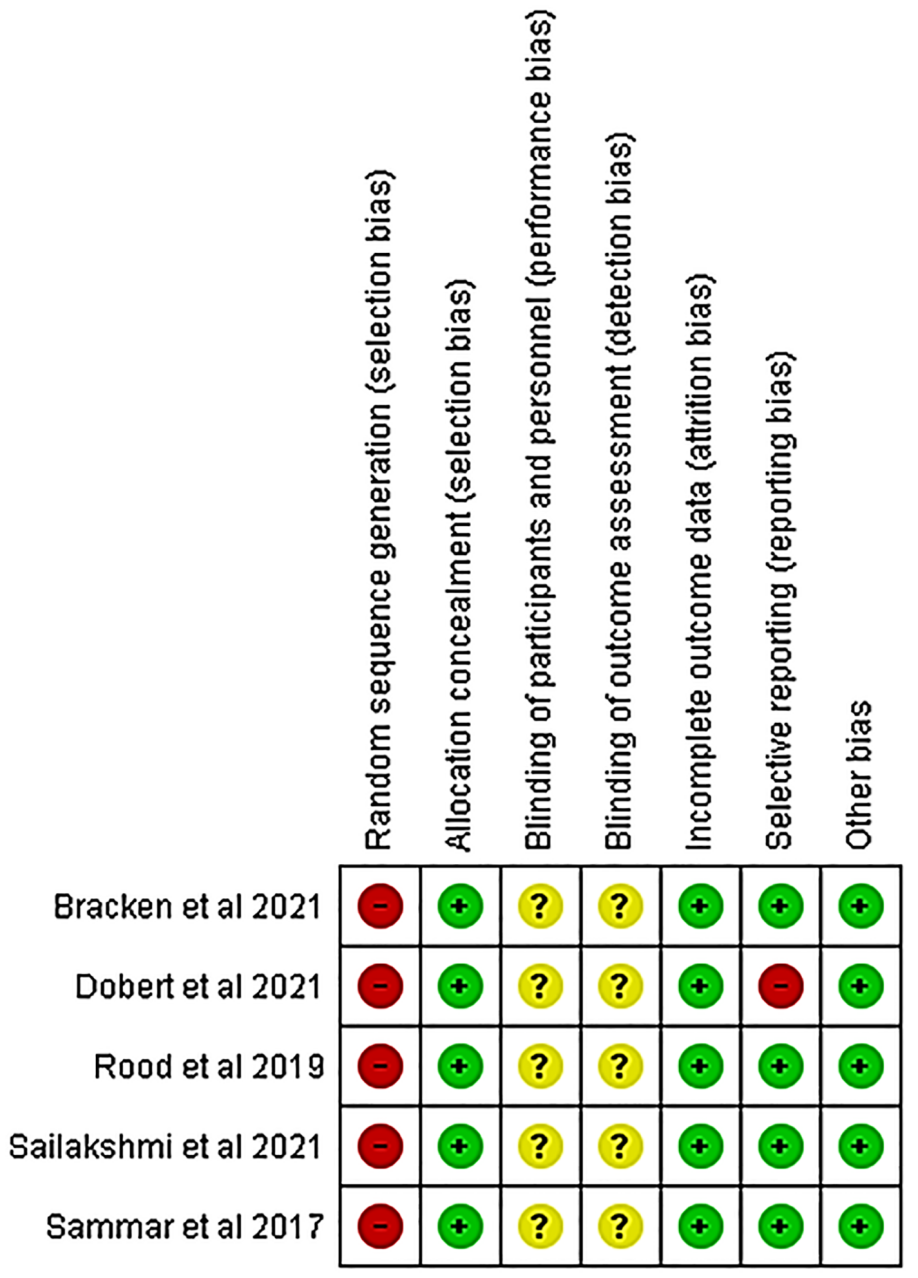
Risk of bias summary review authors' judgments about each risk of bias item for each included study.

**Figure 6:**
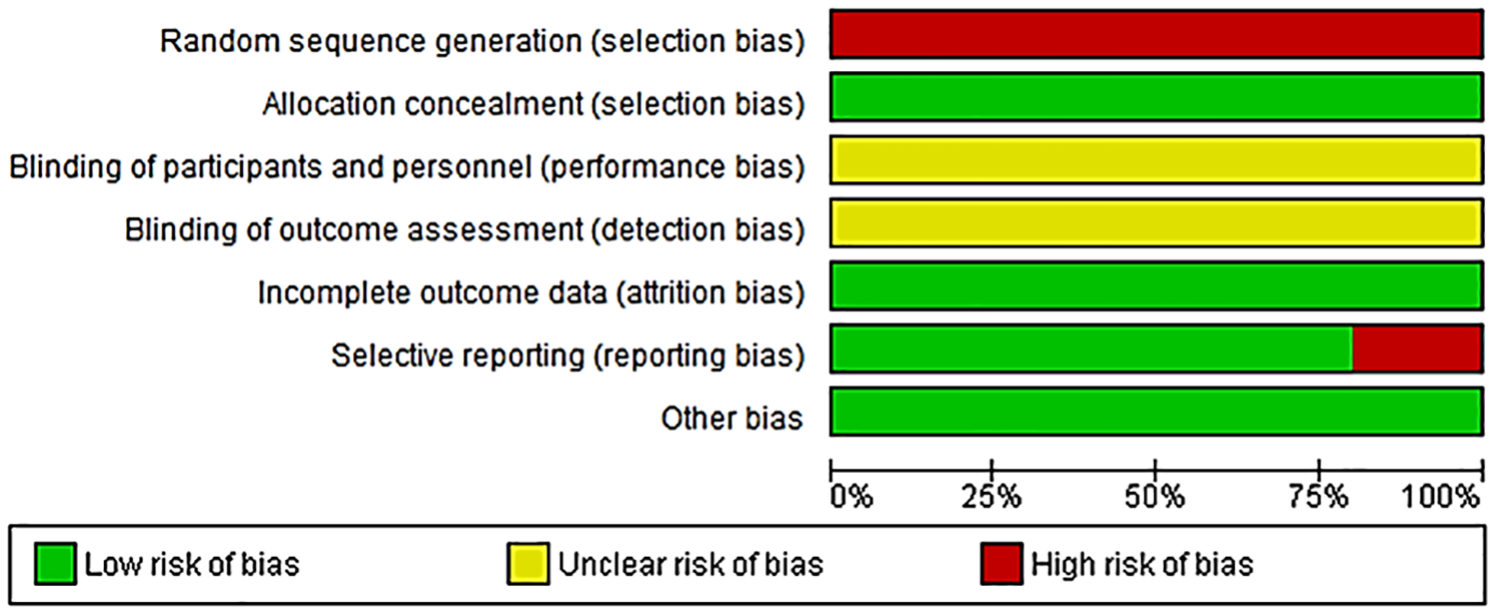
Risk of bias graph about each risk of bias item presented as percentages across all included studies.

Details of the quality of bias assessment are presented in Figures 5, 6. The random allocation was classified as high risk in about 6 studies. All studies were classified as low risk for allocation concealment. Blinding of participants and personnel was classified as unclear in all studies. The studies were classified as low risk for attrition bias. Blinding of outcome assessment (detection bias) was unclear in all studies. There was no evidence of selective reporting (reporting bias) in any of the studies.

## 11. Discussion

In this systematic review and meta-analysis, we examined the effectiveness of CRDPT in the diagnosis of HDP. Our findings showed that CRDPT is not effective in the diagnosis of HDP (Figure 2). Our findings are in support of Dorbert et al., who reported that the performance of CRDPT in diagnosing PE is poor (27). Furthermore, a study by McCarthy et al*.* that evaluated urinary congophilia levels in women with PE, chronic kidney disease without PE, and lupus nephritis in non-pregnant women, confirmed that the CRDPT cannot differentiate between these conditions, suggesting that CRDPT is not a reliable tool in the prediction of PE or HDP (26).

Even though there are studies which found CRDPT to be effective in the detection of PE (21, 22, 30–32), most of these studies had sampling bias (21, 22, 31, 32), whereby higher sampling probability in the PE group was noted compared to the normotensive group (21) or higher sampling probability in the normotensive group compared to PE group (22, 31, 32) (Figures 5, 6). In addition, most of these studies included a mixture of ancestry, mostly Caucasian, and Asian, and a minority of the groups were women of African ancestry (Table 2). In addition, the included studies had a high nature of heterogeneity (*I*^2^ = 97%, *p* < 0.00001) because of the different countries that explored the CRDPT. However, none of these were African countries where HDP is prominent. Furthermore, most of the included studies were cohort studies.

Disorders of pregnancy due to hypertension are one of the causes of maternal morbidity and mortality in LMICs (33). The prevalence of these disorders is higher in women of African ancestry than their counterparts (34). Women of African ancestry in LMICs, mainly in the African continent experience HDP more often than women of a different race and geographic location (high-income-countries). The screening and the detection of hypertension in pregnancy has been difficult in these countries for various reasons like poor infrastructure and equipment. Pre-eclampsia for instance, affects the placenta and the mother systemically leads to more than just elevated blood pressure and protein in the urine, sometimes presenting with hypertension in fusion with by other disorders involving the liver the brain, and thrombocytopenia, resulting in pernicious complications for the mother and her unborn baby (11) such as placenta hypoxia, placental ischemia, and foetal growth restriction.

Misfolded proteins (Amyloid) arise from hypoxia, placental ischemia and pro-inflammatory cytokines leading to endoplasmic reticulum stress in the placenta during pregnancy. These proteins have a high affinity for congophilia and can be detected in the placenta and in circulation in women with PE (35, 36), hence the establishment of the CRDPT to detect these proteins in PE (17). Different tests were used to confirm that the presence of misfolded proteins may be used as a predictor of PE. A11 and polyclonal aAPF antibodies were used to detect amyloid-protofibrils and prefibrillar oligomers in urine specimens of women with PE. Interestingly, these fibrils were not found in women with CH and women without PE (17). In addition, Buhimschi et al. used the CRDPT in women > 34 weeks of gestation to detect misfolded proteins to diagnose PE and as an indication for delivery. The use of the CRDPT proved significant in the study (17). However, another study conducted in China reported a statistically higher detection rate in early-onset (28 weeks to <34 weeks) compared to late-onset (ref 38). This included a cohort of PE women (*n* = 140) and had no control group. Of these PE women, the CRDPT detected misfolded proteins in 83% of severe PE cases, 86% of preterm births, 49% of mild PE cases and 50% at term (37). The findings of this study correspond to that of Dorbet et al. who reported that the CRDPT was a poor predictor of PE in women >35 weeks (27).

Rodriguez et al. used the CRDPT to determine proteinuria in 50 Brazilian women with PE (38). The findings indicated that the dye had high beta protein selectivity and was directly related to proteinuria. The authors concluded that the CRDPT is effective in diagnosing PE, simple, fast and affordable to use in LMICs (38) Furthermore, Sammar et al. used the CRDPT to prediction PE in 81 women from Israel and 642 women from the UK with a gestational age of 26–41 weeks (31). The test proved effective with a higher odd ratio (OR) in obese women and women with a high mean arterial pressure (MAP) and lower in women of African ancestry and a previous history of PE. An adjusted OR of all the parameters was calculated as 13.92 (*p* < 0.001) to predict PE. The study concluded that the CRDPT can be used to predict PE and has been reported as accurate in obese, women, women with high MAP, African ancestry and a previous history of PE (31). These findings contradict our findings because Sammar et al. had a larger population group of women with African ancestry and had no control group (healthy pregnant women) to compare with (31).

Sergeeva et al. conducted a study in Russian women with severe PE (28–36 weeks of gestation). The CRDPT was able to detect misfolded proteins in these women and concluded that the test may be used as a predictor of PE (39). However, this study did not investigate the effectiveness of the CRDPT in pregnant healthy women compared to women with other HDP.

In the current study, Table 2 includes all the countries that used the test and none of the studies were conducted in Africa. Notably, research conducted on the efficacy of the CRDPT globally are limited as the articles found were only twenty and were further reduced to suit the criteria of the research question. Interestingly, as far as we are aware, there are no studies conducted in Africa on the effectiveness of CRDPT in Africa. These findings require confirmation in LMICs where most of the population is of African Ancestry like South Africa.

South Africa is a LMIC and yet faces a high incidence of HDP due to several factors including poor infrastructure, poor health care services, a high prevalence of HIV and delayed hospital care for pregnant women in rural settlements (40). The CRDPT would be of critical importance in SA due to the urgent need to screen, diagnose, and treat HDP early in pregnancy.

The current study has few limitations, there is few studies published that evaluated the accuracy of CRD test in HDP, the included studies had different study designs, sample size, risk of bias and were conducted from different countries. Noteworthy, form of bias that may arise due to poor study methodology. We also noted a very significant rate of high heterogeneity among the included studies, which may be due to the patient characteristics, and samples. However, we evaluated and explored this by performing subgroup and sensitivity analyses, which showed minor significant changes from the initial analysis. Sensitivities and specificities were performed, but these statistics depend on the populations studied, the reference tests used. Quality of studies was analysed using the Cochrane risk of bias.

## 12. Conclusion

This study concludes that the CRDPT proved to be ineffective in the studies presented in the forest plot (Figure 2) due to bias noted in five of the six studies. Furthermore, the studies had larger normotensive groups compared to HDP. Hypertensive disorders of pregnancy are more common in women of African Ancestry. However, the ethnic groups included in this study had a minority of women of African ancestry. Therefore, more research in African countries with a higher prevalence of HDP is required to confirm the effectiveness of the CRDPT.

## Data Availability

The original contributions presented in the study are included in the article/Supplementary Material, further inquiries can be directed to the corresponding author/s.
